# Confronting the surge of Mpox in West Africa

**DOI:** 10.11604/pamj.supp.2025.50.1.49552

**Published:** 2025-10-22

**Authors:** Mat Lowe

**Affiliations:** 1 African Population and Health Research Center, West Africa Regional Office, Dakar, Senegal

**Keywords:** Mpox, West Africa, outbreak

Mpox, also known as monkeypox, is a deadly zoonotic disease. The first human case of Mpox was identified in the 1970s [1]. The disease, however, did not receive much attention until it was declared a Public Health Emergency of International Concern (PHEIC) by the WHO in July 2022 [1]. Since then, it has continued to spread globally, with nearly 93,000 laboratory-confirmed cases and 171 deaths reported from 116 countries as of September 2022. In Africa alone, 2,126 cases and 22 deaths have been reported [1]. In West Africa, the recent surge in the West African clade II Mpox is a significant concern, prompting governments to strengthen their surveillance systems and adopt various response measures.

In Gambia, for instance, the Ministry of Health, through the National Incident Management Team, has partnered with relevant public and private stakeholders to raise public awareness about Mpox and promote preventive measures, including personal hygiene and sanitation, to stop the chain of transmission [2]. The Ministry of Health of Senegal has also collaborated with the Pasteur Institute of Dakar to implement an integrated sentinel surveillance system that involves the collection of real-time data for early detection and case definition [3]. In Sierra Leone, where cases of the West African clade II strain are more pronounced, with more than 3,900 infections and 20 deaths [4], the government has activated emergency mechanisms that facilitate active surveillance, case search, and border screening [5]. The surveillance systems of Guinea and Liberia have also been strengthened, including active contact tracing and case finding, with strategic vaccination efforts targeting high-risk populations in Guinea [6,7]. Despite these measures, responding to the surge of Mpox in these West African countries remains a daunting task.

A significant challenge facing these countries is the weakness of their public health laboratories. Public health laboratories in large parts of these West African countries are weak and severely underfunded [8]. As such, they may not be able to respond to cases of Mpox because they lack modern diagnostic tools for timely and accurate case detection and definition. The porous borders between countries are another challenge. Countries in West Africa have unofficial points of entry (PoEs), which can make the screening of suspected individuals at these points for possible Mpox cases rather challenging. In addition, although the rollout of Mpox vaccines targeting high-risk groups has begun in some countries, such as Guinea, suspicion and mistrust about vaccines in general among the general population can significantly hinder the effective delivery of the Mpox vaccine.

To effectively confront the surge in Mpox in West Africa, there is, therefore, an urgent need to invest in national public health laboratories. This investment should include the purchase of modern diagnostic tools and ensure that health professionals are trained in their use to provide accurate, yet timely, diagnoses, case definitions, and monitoring. Limited diagnostic capacity and skills were major challenges during the early days of the COVID-19 response and continue to be a challenge in confronting the surge of Mpox in West Africa. There is also a need to strengthen border surveillance through regional cross-border cooperation in disease surveillance systems. This will ensure that cases of Mpox are detected and that information is communicated across administrative and regional borders before the disease spreads. Ensuring public trust in information about the Mpox vaccine is another way to confront the spread of Mpox. Lessons from the recent COVID-19 pandemic and the 2014 Ebola epidemic taught us that poor acceptance and mistrust among people and communities contributed immensely to the escalation of the Ebola epidemic and the high rates of mortality in countries such as Guinea, Liberia, and Sierra Leone [9]. Therefore, confronting the surge in Mpox in West Africa will require ensuring trust in information about the Mpox vaccine through sensitization and community engagement and discussion to reach at-risk populations, as well as recruiting and training health professionals to effectively administer the vaccine roll-out [10].
